# Immediate effects of Aussie Current on chronic low back pain: a randomized controlled trial

**DOI:** 10.31744/einstein_journal/2025AO0222

**Published:** 2025-07-23

**Authors:** Leticia Bobato, Gabriele Bressan, Lucas Vinicius Dias, Ramon Schmidt Sale, Audrin Said Vojciechowski, Luiza Helena Gonçalves, Ana Carolina Brandt de Macedo

**Affiliations:** 1 Universidade Federal do Paraná Curitiba PR Brazil Universidade Federal do Paraná, Curitiba, PR, Brazil.; 2 Universidade Positivo Curitiba PR Brazil Universidade Positivo, Curitiba, PR, Brazil.

**Keywords:** Transcutaneous electric nerve stimulation, Low back pain, Pain measurement

## Abstract

**Objective:**

To analyze the immediate analgesic effects of Aussie Current on chronic low back pain using different parameters.

**Methods:**

A total of 105 patients (aged 18-80 years, of both sexes, with chronic low back pain) were randomized into five groups: AG1kHz/100Hz, AG1kHz/2Hz, AG4kHz/100Hz, AG4kHz/2Hz, and placebo. All participants underwent a single application of the Aussie Current for 30 min. The assessments were conducted before and immediately after the intervention, with the following outcomes: pain intensity using the numerical pain rating scale, McGill Pain Questionnaire, mechanical pain threshold, and five-times-sit-to-stand test before and immediately after the intervention. The Start-Back Questionnaire was administered before the intervention to analyze the physical and psychosocial factors related to chronic lower back pain.

**Results:**

In the intragroup analysis, all groups showed significant differences in the numerical pain rating scale and total McGill Pain Questionnaire index. For the mechanical pain threshold, a significant difference was observed in the AG1kHz/100Hz Group at three points in the lumbar region and in the five-times-sit-to-stand test at AG1kHz/100Hz, AG1kHz/2Hz, and AG4kHz/100Hz. In the intergroup comparison, there was a significant difference in the numerical pain rating scale scores between the AG1kHz/100Hz and AG1kHz/2Hz Groups in the Placebo Group.

**Conclusion:**

Aussie Current provides an immediate analgesic effect in individuals with chronic low back pain; however, there is no consensus on the ideal parameters.

**ClinicalTrials.gov Identifier:** RBR-98HJ9X.

## In Brief

A total of 105 patients with chronic low back pain of both sexes underwent a single application of Aussie Current for 30 min and were randomized into five groups: AG1kHz/100 Hz, AG1kHz/2 Hz, AG4kHz/100 Hz, AG4kHz/2 Hz, or placebo. Pain intensity was assessed in all individuals. The Aussie Current provides an immediate analgesic effect in individuals with chronic low back pain, but there has been no conclusion on the ideal parameter.

## Highlights

■ Aussie Current decreases immediate pain intensity.
■ Both carrier frequencies of Aussie Current were efficient in decreasing pain.
■ There are no ideal parameters for using the Aussie Current to relieve pain in individuals with chronic lower backpain.

## Graphical abstract

**Figure:**
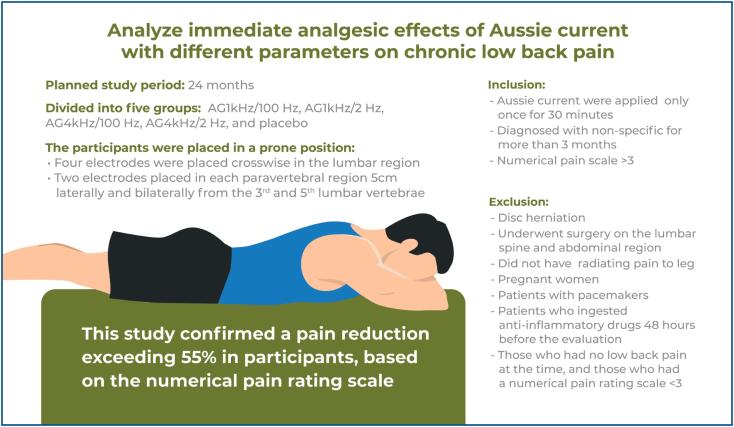

## INTRODUCTION

Low back pain (LBP) is among one of the most common musculoskeletal complaints, affecting up to 84% of the population worldwide at some point in their lives.^(1)^ In most cases, the pain disappears within six weeks, whereas in other cases, there is no improvement, and the pain progresses to a chronic condition.^(2)^ It is often difficult to determine the exact cause of LBP because of its multifactorial origins, including sociodemographic and biopsychosocial factors, profession, and lifestyle.^(3)^ Patients with chronic LBP experience considerable pain and impaired functional capacity daily, which leads to work absences and harms both the individual and society economically.^(1,4)^

Thus, given the health and economic spheres, it is important to seek an effective treatment for nonspecific chronic low back pain (CLBP) that is non-invasive, non-pharmacological, and inexpensive. Electrotherapy is an indicated physical therapy resource because it is cheaper than surgical and pharmacological therapy. In addition, electrotherapy mediates its effects via electrical stimuli. This can activate the nervous system fibers.^(5-7)^ Among the currents used in electrotherapy are low-frequency currents, such as transcutaneous electrical nerve stimulation (TENS), and medium-frequency alternating currents, such as interferential, Russian, and Aussie currents (AC). AC, a medium-frequency alternating current (1 or 4kHz) modulated at low frequencies (1 and 120Hz), differs from other currents because it has a short burst duration, which makes it more comfortable.^(8)^

Previous studies have indicated that AC can produce torque and increase muscle strength;^(8,9)^ a pulse frequency of 1kHz and bursts with a duration between 2 to 4ms are considered necessary to produce torque. Regarding its analgesic potential, some researchers^(10,11)^ have found that AC is as effective as TENS in pain relief.^(8,11)^ For pain relief, a pulse frequency of 4kHz, with a burst of 4ms, is recommended.^(11)^

According to Imamura et al.,^(12)^ individuals with LBP have increased excitability by 28 in the central nervous system, indicating the amplification of nociceptive processes 29 and demonstrating impaired conditioned pain modulation. The analgesic effect of AC is 30 like that of TENS, although their physical properties differ. In animal studies, 31 TENS has been found to reduce central excitability by activating 32 central inhibitory pathways.^(13,14)^ The gate control theory of pain is the commonly used theory to 33 explain pain inhibition by TENS. According to this theory, stimulation of large 34 diameter afferents by TENS inhibits nociceptive fibers and evokes responses in the spinal cord's dorsal horn.^(15)^ Moreover, TENS activates the opioid receptors in the central 2 nervous system, inducing analgesia.^(16)^

Silva et al.^(17)^ used AC in individuals with chronic nonspecific neck pain for analgesia (4kHz, 5Hz, and 4ms) over 12 sessions; however, there was no significant improvement in pain or functionality. Ward et al.^(9)^ compared the analgesic effects of TENS (50Hz) and AC (4kHz, 50Hz, 4ms) in healthy individuals and found that AC provided greater comfort on application with no significant difference in the analgesic effect. Rampazo et al.^(18,19)^ compared the analgesic effects of different electrical currents (TENS, Interferential Current, and AC) on the pain and comfort thresholds of healthy individuals and found no significant differences.

To the best of our knowledge, no previous study has evaluated the analgesic effect of AC in individuals with CLBP or compared the effects of different pulse frequencies and frequency modulations. Only one study^(20)^ verified the analgesic action of AC applied to strengthen the lumbar spinal erectors (1kHz, 50Hz, 4ms) during 12 sessions (4 weeks) and found improvement in pain and lumbar muscle resistance.

## OBJECTIVE

To analyze the immediate effect of the Aussie Current with different application parameters on the subjective perception and maximum tolerance of mechanical pain in individuals with chronic low back pain, and to assess functionality.

## METHODS

### Study design

This was a double-blind, five arms, controlled, randomized clinical trial. This study was approved by the Ethics and Research Committee of the *Universidade Federal do Paraná* (CAAE: 44642615.2.0000.0102; #1.145.540).

### Study location

Data were collected at the Physiotherapy Laboratory of the *Universidade Federal do Paraná* between August 2018 and September 2019.

### Eligibility criteria

The inclusion criteria were individuals between 18 and 85 years of age of both sexes, with nonspecific CLBP (more than 12 weeks),^(3)^ pain intensity using the numerical pain rating scale (NPRS) greater than 3.^(21)^ Those who agreed to participate after a verbal invitation signed a Free and Informed Consent Form (Resolution 466/2012 of the National Health Council). Nonspecific pain, which is caused by pain (such as infection, neoplasm, metastasis, osteoporosis, rheumatoid arthritis, or inflammatory processes), was not identified.^(3)^

The exclusion criteria were patients with disc herniation or who underwent surgery on the lumbar spine and abdominal region, did not have radiating pain in the leg, were pregnant, had pacemakers, ingested anti-inflammatory drugs 48 hours before the evaluation, had no low back pain at the time, and had an NPRS< 3.

### Sample calculation

The sample size was calculated using G*Power3.1.9.4, assuming a difference of two points in pain intensity using the NPRS, with an estimated standard deviation of 1.47 points. Considering a test power of 80%, significance level of 5%, and sample loss of 10%, 23 participants were recruited for each group (115). In addition, owing to sample loss, the power of the sample was 0.71 with 105 participants.

### Randomization

Randomization was performed using six blocks. Five blocks contained 20 papers, four of which were from each group: AG1kHz/100Hz (n=4), AG1kHz/2Hz (n=4), AG4kHz/100Hz (n=4), AG4kHz/2Hz (n=4), and Placebo Group (PG) (n=4). The last block contained 15 papers, with three papers from each group, for a total of 115 papers. Each block was randomized using a raffle.^(22)^ A new block was randomized only after the completion of the previous block; that is, after 20 papers from that block were selected. The selection process was blinded to both the participants and evaluators, with a third person responsible for controlling the process.

### Intervention

The participants were randomized into five groups: AG1kHz/100Hz, AG1kHz/2Hz, AG4kHz/100Hz, AG4kHz/2Hz, and PG Groups.

For AC application, the participants were placed in a prone position, and four electrodes were placed crosswise in the lumbar region, with two electrodes placed in each paravertebral region 5cm laterally and bilaterally from the 3^rd^ and 5^th^ lumbar vertebrae.^(23)^ The electrodes were made of silicone (90mm × 50mm) with a conductive gel and fixed using adhesive tape. We used the same application method as the TENS. The Aussie equipment used was Neurodyn (IBRAMED), previously calibrated, with the application of 30 min of AC. The parameters for each current were as follows.

– AG1kHz/100Hz: pulse frequency (PF)=1kHz, modulated at 100Hz, burst=4ms, intensity (I)=sensory level.
– AG1kHz/2Hz: PF=1kHz, modulated at 2Hz, burst=4ms, I=motor level.
– AG4kHz/100Hz: PF=4kHz, modulated at 100Hz, burst=4ms, I=sensory level.
– AG4Hz/2Hz: FP=4kHz, modulated at 2Hz, burst=4ms, I=motor level.

The PG procedure mirrored others, however, intensity remained unchanged; no parameters were applied. The therapist asked the participants whether they could feel the sensation of the current. All groups received this command; the PG showed no response.

Only one application of the Aussie Current was performed, and reassessment was performed immediately after 30 min.

### Evaluated clinical outcomes

A single blinded researcher assessed participants before and directly after the intervention. The researcher was uninvolved in the study's application, remaining unaware of participant group assignments. Second Applied Current. Participants were assessed using a specific form containing data for the identification, anamnesis, assessment, and classification of pain using scales (NPRS and McGill questionnaire) and mechanical pain tolerance (using pressure algometry). Functional tests (five-times-sit-to-stand test [5XSST]) were also performed, and the Start-Back Questionnaire (SBST) was used to assess biopsychosocial factors associated with LBP. These instruments were used before and 30 min post-AC, AC preceding the current application.

The NPRS is a 10cm long horizontal line numbered from 0 to 10, with 0 indicating no pain and 10 indicating the maximum pain. The participants indicated the point representing the intensity of their pain at the time of assessment.^(24)^ Pain was classified according to Boonstra et al.^(21)^ and categorized as low (NPRS<3), moderate (NPRS between 4 and 6), or high (NPRS≥7).

The McGill Pain Questionnaire (MPQ), validated in Portuguese by Mercedes Costa et al.,^(25)^ has 0.46 to 0.80 reliability and good construct validity. It assesses several aspects of pain using the words (descriptors) that participants choose to express their pain. The 78 descriptors that qualified for pain were divided into four categories: sensory discriminative, emotional motivational, cognitive, and miscellaneous, and into 20 subcategories. Participants could choose a word per subcategory, or choose none. The numerical index of the descriptors was calculated as the number of words chosen by the participants to characterize their pain, with a maximum of one word from each subgroup and a maximum of 20 words. A numerical index was calculated for each category.^(25)^

Mechanical pain tolerance (MPT) was assessed using an algometer (EMG System, Brazil). To perform the algometer evaluation, four points were demarcated in the lumbar region (5cm from the third and fifth lumbar vertebrae on both the right and left sides).^(23)^ In addition, points were marked for use as controls: the midpoint of the tibial muscle, anterior to 5cm, lateral to the tibial tuberosity of the right and left legs. The tip of the algometer (1cm in diameter) was pressed at each point perpendicular to the participant's skin, and the participant was instructed to say "stop" when they could no longer tolerate the pressure; the pressure was recorded in the evaluation form. Averaging three samples from each point determined MPT. Each collection was conducted in sequence: first to the left (L) of L3, and then to the right (R) of L3, left of L5, and right of L5.^(23)^ The development rate was 0.3 Kgf/s, and the second collection was started at the end of the application at the last point. We evaluated the resource both immediately before and after its 30-min application. To calculate the intraclass correlation coefficient, the researcher evaluated it within an interval of 48hours. Data analysis indicated excellent reliability of the interventionist's results (intraclass correlation coefficient, 0.95).

The Start-Back Questionnaire (SBST) assesses physical and psychosocial factors and relates them to possible prognoses in individuals with LBP. It has a reliability of 0.79 and good validity.^(26)^ The SBST comprises nine items, four of which are related to pain, functionality, and comorbidities, and five of which are related to socio-psychological factors. Individuals were classified as high risk (greater presence of psychosocial factors than physical factors), medium risk (low presence of psychosocial and physical factors compared to high risk), or low risk (minimal presence of psychosocial and physical factors); a total score between 0 and 3 was considered low risk. For a final score above three, psychosocial factors were considered, and the number of questions was selected from five to nine. If the total was ≤3, individuals were classified as medium risk, and if the total was ≥3, individuals were classified as high risk.^(26)^

The five-times sit-to-stand test (5XSST) assesses the strength and muscle performance of the lower limbs. During the test, the individual was seated in a chair with their feet resting on a flat surface, an erect spine, and crossed upper limbs. Under command, the subject was instructed to perform five sit-to-stand as quickly as possible without the aid of the upper limbs during the test. The time was measured using a stopwatch. The test was performed three times with an interval of 1 min between each repetition, and the average of the three repetitions was calculated.^(27)^

### Statistical analysis

The parameters were analyzed using SPSS Software version 25.0. The results are expressed as mean±standard deviation and were subjected to analysis of normality and homogeneity of variances using the Shapiro-Wilk and Levene tests, respectively. We conducted a prospective, intention-to-treat analysis of the data. For parametric variables, an analysis of covariance (ANCOVA) of repeated measures was performed for intergroup comparisons, with the SBST as the covariable. For non-parametric variables, the Wilcoxon test was used for intragroup analysis and the Kruskal–Wallis test for intergroup analysis. The effect size (ES) (Cohen's d) was estimated using the mean of the differences between the groups, in which 0.1, 0.25, and 0.40 were considered small, medium, and 0.40 as a large effect, respectively. Statistical significance was set at p<0.05 for statistical significance.

## RESULTS

**Figure 1:**
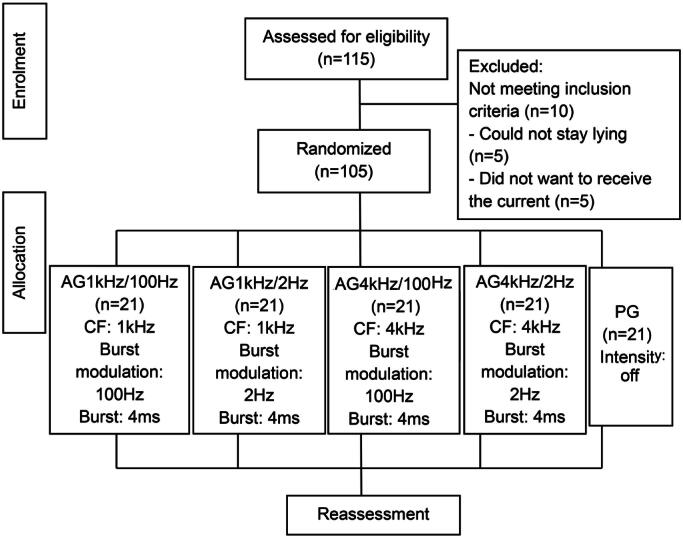
Study design

AC: acoustic current; CF: carrier frequency; y: time; PG: Placebo Group.

A total of 115 patients were evaluated and divided into five groups: AG1kHz/100Hz, AG1kHz/2Hz, AG4kHz/100Hz, AG4kHz/2Hz, and PG Groups. The study had a sample loss of 10 individuals (Figure 1), leaving 105 participants.

Table 1 shows the sociodemographic and clinical characteristics of the study population. The mean age was 29.9±12.5 years, with a predominance of females (n=56), incomplete college education (n=60), non-smokers (n=96), non-alcoholics (n=87), and non-sedentary lifestyles (n=63). Regarding pain location, most participants reported centralized pain (n=46) that worsened at night (n=41) and during effort (n=97), Regarding biopsychosocial factors, 44 were considered high risk, 12 medium risk, and 49 low risk.

**Table 1:** Clinical and sociodemographic characteristics

		AG1kHz/100Hz (n=21)	AG1KHz/2Hz (n=21)	AG4KHz/100Hz (n=21)	AG4KHz/2Hz (n=21)	PG (n=21)	p value
Age (mean±SD)		33.4+17.1	28.3±10.5	35.5±16.6	23.8±7.9	28.9±10.8	>0.05
Gender, n (%)							
	Female	13 (61.9)	9(42.9)	10 (47.6)	12 (57.1)	12(57.1)	
	Male	8 (38.1)	12 (57.1)	11 (52.4)	9(42.9)	9(42.9)	
Scholarity, n (%)							>0.05
	Incomplete fundamental	1 (4.8)	1 (4.8)	0 (0)	0 (0)	0 (0)	
	Complete fundamental	2 (9.5)	0 (0)	0 (0)	0 (0)	0 (0)	
	Incomplete high school	0 (0)	0 (0)	1 (4.8)	2 (9.5)	0 (0)	
	Complete high school	2 (9.5)	1 (4.8)	6 (28.6)	0 (0)	4 (19)	
	Incomplete college	11 (52.4)	14 (66.7)	8 (38.1)	17(81)	10 (47.6)	
	Complete college	5 (23.8)	5 (23.8)	6 (28.6)	2(9.5)	7 (33.3)	
Life habits, n (%)							
	Smoker	2 (9.5)	0 (0)	1 (4.8)	1 (4.8)	5 (23.8)	
	Alcohol consumption	3 (14.3)	1 (4.8)	2 (9.5)	9(42.9)	3 (14.3)	
	Sedentary	11 (52.4)	8 (38.1)	10 (47.6)	4 (19)	9(42.9)	
Time of pain (months) (Mean. min. max. median)		3.8;1;10;2	7.3;1;60;3	5.8;1;20;3	3.6;1;10;3	3.1;1;6;3	>0.05
Location of pain, n (%)							
	Centralized	9(42.9)	9(42.9)	9(42.9)	6(28.6)	13(61.9)	
	At the right	5 (23.8)	0 (0)	6 (28.6)	4 (19)	3 (14.3)	
	At the left	2 (9.5)	4 (19)	3 (14.3)	3 (14.3)	2 (9.5)	
	Bilateral	5 (23.8)	8 (38.1)	2 (9.5)	8 (38.1)	3 (14.3)	
Period of the day when pain worsens, n (%)							
	Morning	7 (33.3)	9(42.9)	8 (38.1)	3 (14.3)	7 (33.3)	
	Afternoon	1 (4.8)	7 (33.3)	4 (19)	8 (38.1)	4 (19)	
	Night	13 (61.9)	5 (23.8)	9(42.9)	10(47.6)	4 (19)	
Activities that exacerbate pain, n (%)							
	Walk	8 (38.1)	5 (23.8)	8 (38.1)	3 (14.3)	9(42.9)	
	Sit	10(47.6)	12 (57.1)	12 (57.1)	11 (52.4)	12 (57.1)	
	Bend	12(57.1)	14 (66.7)	14 (66.7)	11 (52.4)	12 (57.1)	
	Get up	13 (61.9)	7 (33.3)	10 (47.6)	11 (52.4)	12 (57.1)	
	Climbing stair	4 (19)	5 (23.8)	7 (33.3)	5 (23.8)	5 (23.8)	
	Effort/lift object	19 (90.5)	20 (95.2)	17 (81)	20 (95.2)	21 (100)	
Pain classification							
	Mild	12(57.1)	19 (90.5)	17 (81)	17(81)	20 (90.5)	
	High	9 (42.5)	2 (9.5)	4 (19)	4 (19)	1 (4.8)	
	Start back						
	Risk lower	6 (28.6)	10 (47,6)	10 (47,6)	12 (57.1)	11 (52.4)	
	Risk medium	4 (19)	2 (9.5)	1 (4.8)	4 (19)	1 (4.8)	
	Rish higher	11 (52.4)	9(42.5)	10 (47.6)	5 (23.8)	9(42.9)	

PG: Placebo Group.

In the intragroup analysis (Table 2), all groups showed a significant difference in the NPRS categories and total MPQ index. AG1kHz/100Hz (p=0.00) and AG1kHz/2Hz (p=0.00) showed the greatest reduction in NPRS (71.7% and 78.7%, respectively), whereas AG4kHz/100Hz and AG4kHz/2Hz exhibited reductions of 55.7% and 62.5%, respectively, and the PG showed a reduction of 38.6%. In the MPT, a significant difference was observed at three points in the lumbar region (RL3, LL5, RL5) of AG1kHz/100Hz (p=0.02); this group also showed significance in the 5XSST (Wilcoxon test, p<0.05), AG1kHz/2Hz, and AG4kHz/2Hz.

**Table 2:** Evaluation of Numerical Pain Rating Scale. McGill Pain Questionnaire and pressure pain threshold (within groups)

mean±SD (min; max; med)	AG1kHz/100Hz (n=21)		AG1kHz/2Hz (n=21)		AG4kHz/100Hz (n=21)		AG4kHz/2Hz (n=21)		PG (n=21)	
	Before	After	Before	After	Before	After	Before	After	Before	After
NPRS	5.3±2.3 (3;8;6)	1.5±1.5 (0;4;2)	4.7±2.0 (3;9;6)	1.0±1.2 (0;4;1)	5.2±1.5 (3;7;6)	2.3±1.8 (0;5;2)	4.0±1.8 (3;8;4)	1.5±1.6 (0;5;1)	4.4±1.0 (3;7;5)	2.7±1.8 (0;7;3)
p value	0.00#		0.00#		0.00#		0.00#		0.00#	
MPQ										
Sensory	8.1±1.8 (4;10;8)	3.5±4.0 (0;10;1)	7.4±2.7 (2;10;8)	2.7±3.9 (0;10;0)	7.2±3.2 (1;10;9)	4.3±4.2 (0;10;3)	7.8±2.0 (2;10;8)	3.8±4.0 (0;10;3)	8.0±2.3 (2;10;9)	5.6±3.5 (0;10;7)
p value	0.00*		0.00*		0.00*		0.00*		0.00*	
Affective	3.1±1.7 (0;5;3)	0.6±1.3 (0;5;0)	3.1±1.7 (1;5;3)	0.7±1.7 (0;5;0)	3.4±1.9 (0;5;5)	1.5±2.0 (0;5;0)	3.1±1.5 (0;5;3)	1.0±1.8 (0;5;0)	3.4±1.3 (0;5;3)	1.4±1.9 (0;5;0)
p value	0.00*		0.00*		0.00*		0.00*		0.00*	
Evaluative	1.0±0.0 (0;1;1)	0.4±0.5 (0;1;0)	1.0±0.0 (0;1;1)	0.3±0.4 (0;1;0)	0.9±0.2 (0;1;1)	0.5±0.5 (0;1;1)	0.9±0.2 (1;1;1)	0.4±0.5 (0;1;0)	0.9±0.2 (0;1;1)	0.7±0.4 (0;1;1)
p value	0.00*		0.00*		0.00*		0.00*		0.02*	
Miscellaneous	2.8±0.9 (1;4;3)	1.0±1.3 (0;4;0)	2.2±1.3 (0;4;2)	0.9±1.3 (0;4;0)	2.9±1.2 (0;4;4)	1.5±1.5 (0;4;1)	2.8±1.5 (0;4;3)	0.8±1.4 (0;4;0)	2.3±1.4 (0;4;3)	1.9±1.7 (0;4;1)
p value	0.00*		0.01*		0.00*		0.00*		0.25	
Total	15.1±3.6 (9;20;15)	5.1±5.9 (0;18;2)	13.8±5.3 (4;20;14)	2.9±5.4 (0;20;0)	14.5±6.4 (2;20;19)	7.5±7.6 (0;20;4)	14.4±4.5 (3;20;14)	5.5±6.9 (0;20;2)	14.8±4.7 (3;20;16)	9.3±7.1 (0;20;9)
p value	0.00*		0.00*		0.00*		0.00#		0.00*	
MPT										
ATL	4.3±1.5 (1.7;8.4;3.8)	4.8±3.2 (0.0;16.1;4.0)	6.4±3.3 (1.4;14.6;6.0)	6.6±2.8 (1.7;13.3;6.7)	6.1±3.1 (1.7;13.7;5.1)	6.1±3.6 (1.6;17.4;5)	5.6±2.5 (2.0;13.1;5.9)	6.1±3.0 (0.1;13.1;6.8)	6.2±2.9 (1.8;12.9;6.4)	6.3±3.2 (1.6;13.3;6.0)
p value	0.17		0.61		0.89		0.17		0.92	
ATR	4.5±2.0 (1.9;9.4;3.9)	4.7±2.6 (0.1;12.5;4.1)	7.0±3.5 (1.6;14.4;7.2)	6.9±3.2 (1.7;14.4;7.0)	6.2±3.7 (1.7;17.7;4.8)	6.0±3.8 (1.5;19.0;4.8)	5.5±2.4 (1.5;10.5;5.5)	5.3±2.5 (0.1;10.8;5.7)	6.2±3.2 (1.9;14.6;6.0)	6.4±3.8 (1.2;16.4;6.4)
p value	0.63		0.68		0.52		0.62		0.77	
LL3	3.7±1.2 (1.4;7.1;3.6)	4.2±1.9 (1.1;8.7;3.7)	4.9±2.6 (1.8;11.8;4.6)	5.2±2.3 (2.0;9.7;4.1)	5.2±2.2 (2.1;9.4;4.7)	5.5±2.3 (2.0;11.7;4.6)	4.3±2.1 (1.5;7.6;4.3)	4.7±1.6 (2.4;7.5;4.2)	4.3±1.7 (1.1;7.8;4.3)	4.1±2.0 (0.9;8.3;4.1)
p value	0.06		0.28		0.43		0.13		0.32	
RL3	3.9±1.4 (0.8;7.2;3.9)	4.5±1.7 (1.1;8.2;4.2)	5.1±2.7 (1.8;12.0;4.7)	5.3±2.2 (1.7;10.3;4.9)	5.4±2.9 (1.3;11.7;4.2)	5.6±2.7 (2.2;12.2;4.8)	4.4±2.3 (1.0;8.5;4.1)	4.5±1.2 (1.9;6.4;4.4)	4.6±2.1 (1.8;10.4;4.2)	4.6±2.5 (1.2;9.9;4.1)
p value	0.04#		0.61		0.48		0.71		0.82	
LL5	3.4±1.1 (1.3;5.9;3.2)	4.0±1.8 (0.9;9.9;3.9)	4.6±2.6 (1.7;13.2;4.6)	4.6±2.0 (1.7;8.2;4.8)	4.9±2.3 (1.6;11.6;4.3)	5.1±2.9 (2.4;14;3.9)	4.8±3.6 (1.7;17.2;3.4)	4.5±1.5 (2.1;7,8;4.70	4.2±1.7 (1.4;7.6;4.1)	4.0±2.0 (1.2;8.6;4.0)
p value	0.02#		0.91		0.79		0.59		0.32	
RL5	3.8±1.6 (1.1;7.5;3.6)	4.3±2.0 (0.8;10.9;3.9)	4.6±2.5 (1.4;12.3; 4.6)	4.9±2.2 (2.0;9.8;4.8)	5.1±2.5 (1.4;12.8;4.5)	5.0±2.3 (2.2;10.9;4.5)	4.5±2.3 (1.2;9.2;4.6)	4.6±1.5 (1.8;6.9;4.7)	4.4±2.3 (1.7;10.5;8.8)	4.5±2.3 (1.2;8.8;3.7)
p value	0.05#		0.29		0.71		0.70		0.5	
5XRSS	15.7±9.1 (9;24;12.3)	19.1±8.2 (9;20;11.3)	19.1±11.4 (9;21;10.8)	21.5±11.9 (8;16;10.2)	23.7±10.0 (8;27;11.7)	22.2±12.3 (0;27;12.0)	23.3±9.8 (8;15;11.4)	24.6±8.5 (0;14;10.1)	23.1±6.5 (8;16;10.6)	21.6±7.5 (0;19;11.0)
p value	0.00*		0.00*		0.06		0.00#		0.09	

* p<0.05 (Wilcoxon test);

# pared test

SD: standard deviation; min: minimum; max: maximum; med: median; NPRS: Numerical Pain Rating Scale. MPQ: McGill Pain Questionnaire; MPT: mechanical pain tolerance; ATL: anterior tibial left; ATR: anterior tibial right.

Table 3 shows the intergroup comparison. In the NPRS, AG 1kHz/100Hz (p=0.06) and AG 1kHz/100Hz (p=0.06) differed from the PG results.

**Table 3:** Between group analysis

Mean±SD (min; max; med)		Groups				PG
		AG1KHz/100Hz	AG1KHz/2Hz	AG4KHz/100Hz	AG4KHz/2Hz PG	
NPRS		5.3±2.3 (1;8;6)*	4.7±2.0 (0;9;6)*	5.2±1.5 (1;7;6)	4.0±1.8 (2;8;4)	4.4±1.0 (2;7;5)
MPQ						
	Sensory	8.1±1.8 (4;10;8)	7.4±2.7 (2;10;8)	7.2±3.2 (1;10;9)	7.8±2.0 (2;10;8)	8.0±2.3 (2;10;9)
	Affective	3.1±1.7 (0;5;3)	3.1±1.7 (1;5;3)	3.4±1.9 (0;5;5)	3.1±1.5 (1;5;3)	3.4±1.3 (0;5;3)
	Evaluative	0.9±0.2 (0;1;1)	0.9±0.2 (0;1;1)	0.9±0.2 (0;1;1)	0.9±0.2 (0;1;1)	0.9±0.2 (0;1;1)
	Miscellaneous	0.2±0.9 (1;4;3)	2.2±1.3 (0;4;2)	2.9±1.2 (0;4;3)	2.5±1.5 (0;4;3)	2.3±1.4 (0;4;3)
	Total	13.4±3.6 (9;20;15)	13.8±5.3 (4;20;14)	14.5±6.4 (2;20;19)	14.4±4.3 (3;20;14)	14.8±4.7 (3;20;16)
MPT						
	ATL	4.3±1.5 (1.7;8.4;3.8)	6.4±3.3 (1.4;14.6;6.0)	6.1±3.7 (1.7;13.7;5.1)	5.6±2.5 (2.0;13.1;5.9)	6.2±2.9 (1.8;12.9;6.4)
	ATR	4.5±2.0 (1.9;9.4;3.9)	7.0±3.5 (1.6;14.4;7.2)	6.2±3.7 (1,7;17.0;4.8)	5.5±2.4 (1.5;10;5;5.5)	6.2±3.2 (1.9;14.6;6.0)
	LL3	3.7±1.2 (1.4;7.1;3.6)	4.9±2.6 (1.8;11.8;4.6)	5.2±2.2 (2.1;9.4;4.7)	4.3±2.1 (1.5;7.6;4.3)	4.3±1.7 (1.1;7.8;4.3)
	RL3	3.9±1.4 (0.8;7.2;3.9)	5.1±2.7 (1.8;12.0;4.7)	5.4±2.9 (1.3;11.7;4.2)	4.3±2.3 (1;8.5;4.1)	4.6±2.1 (1.8;10.4;4.2)
	LL5	3.4±1.1 (1.3;5.9;3.2)	4.6±2.6 (1.7;13;2;4.6)	4.9±2.3 (1.6;11.6;4.3)	4.8±3.6 (1.7; 17.2;3.4)	4.2±1.7 (1.4;7.6;4.1)
	RL5	3.8±1.6 (1.1;7.5;3.6)	4.6±2.5 (1.4;12.3;4.6)	5.1±2.5 (1.4;12.8;4.5)	4.5±2.3 (1.2;9.2;4.6)	4.4±2.3 (1.7;10.5;3.7)
	5XRSS	1.3±2.3 (-2;7;1)	0.8±1.0 (0;4;1)	0.8±2.3 (-5;7;1)	0.7±1.0 (-2;2;1)	0.1±1.3 (-3;3;0)

* p<0.05, compared to placebo (Kruskal-Wallis test).

SD: standard deviation; min: minimum; max: maximum; med: median; NPRS: Numerical Pain Rating Scale. MPQ: McGill Pain Questionnaire; MPT: mechanical pain tolerance; ATL: anterior tibial left; ATR: anterior tibial right.

## DISCUSSION

This study shows that the use of AC plays a role in the management of CLBP due to its immediate analgesic effect, and a PF of 1 KHz obtained significant results in the PG. Thus, AC can be considered a complementary therapy before initiating kinesiotherapy.

The SBST, of which 46.6 presented low risk, 11.4% medium risk, and 41.9% high risk, was used to assess the influence of biopsychosocial factors associated with CLBP. When this factor was used as a covariate for pain outcomes using the NPRS, MPQ, and MPT, no significant differences were observed. Therefore, it can be concluded that biopsychosocial factors did not influence the study results. However, when the covariate pain classification was inserted, significant differences were found in pain, the higher the classification, the greater the improvement.

This study confirmed a pain reduction exceeding 55% in participants, based on the NPRS. Ostelo et al.^(28)^ reported that a decrease of >30% in posttreatment NDT represented a minimally important clinical change. The intervention groups showed differences greater than 3 points compared to the PG. According to Chou et al.,^(29)^ this indicates a strong treatment effect. These results corroborate those of Pelegrini et al.,^(20)^ who applied AC (1kHz, 50Hz, 4ms) to strengthen the lumbar region, resulting in a significant decrease in pain, as measured using the Visual Analog Scale (VAS), after 12 treatment sessions. However, Silva et al.^(17)^ applied AC (1kHz, 50Hz, 4ms) to individuals with neck pain over 12 treatment sessions and found no significant difference in pain using the VAS. Rampazo et al.^(18,19)^ used AC (4kHz, 100Hz, 4ms) after 30 min compared to other forms of electrostimulation (Interferential Current (IFC) and TENS) and failed to conclude that AC was superior regarding VAS, although similar sensory comfort was shared between them.

Although scientific findings on this subject are limited, the use of ACs in rehabilitation is increasing worldwide. Another medium-frequency electrical current is the IFC, which has undergone more clinical trials, although the best parameters remain under investigation. Albornoz-Cabello et al.^(30)^ showed the advantage of using IFC with a 4kHz carrier frequency (CF) with 65Hz modulation in the short-term VAS. Lara-Palomo et al.^(31)^ used the same CF (4kHz) and AMF (80Hz) and found significant intergroup differences in VAS scores after 20 sessions. In this study, Corrêa et al.^(32)^ Almeida et al.^(33)^ and Almeida et al.^(23)^ analyzed the immediate analgesic effect and showed a significant decrease in the VAS score after treatment with IFC using a CF of 1kHz and 4kHz, with an AMF of 100Hz and 2kHz.

This study revealed significant post-intervention improvements across all MPQ domains for every group, except for the miscellaneous PG intragroup. Using the IFC, Facci et al.^(34)^ also showed improvement in the intervention with the MPQ indexes, albeit using a 4kHz CF with an amplitude modulation of 20Hz. Almeida et al.^(23)^ demonstrated a significant improvement in the MPQ with an IFC of 4kHz and an AMF of 100Hz. Pelegrini et al.^(20)^ also found a satisfactory improvement in the total MPQ index after the application of 12 sessions of AC (1kHz, 50Hz, and 4ms) for lumbar strengthening, which agrees with the results of our study, in which the group with a PF of 1kHz showed a significant difference from the PG.

The results of the objective measurement of pain using the algometer indicated that AG1kHz/100Hz showed a significant difference at the three points in the lumbar region. Almeida et al.^(33)^ and Almeida et al.^(23)^ reported significant results when using IFC with a higher base frequency (4kHz) and 100Hz modulation. Similarly, Rampazo et al.^(19)^ showed that the pain threshold pressure increased in relation to PG after the application of AC, whereas in the other groups (AG 1kHz/2Hz, AG 2kHz/100Hz, and AG 4kHz/2Hz), no increase in MPT was observed. This agrees with the study by Almeida et al.,^(23)^ who used IFC.

According to Colloca et al.,^(35)^ placebo effects have been attributed to expectations. This theory assumes that the placebo produces an effect because the recipient expects it. In this study, despite the PG showing a decrease in pain, as observed in the 14 NPRS after treatment (intragroup), there was a greater improvement in the 2kHz/100Hz group than in the PG. Regarding the MPQ, the AG 1kHz/2Hz group showed significant improvement in the sensory subcategories and total index compared to the PG.

Regarding the 5XSST results, our study indicated that the groups that improved after the intervention were the AG 1kHz/100Hz, AG 1kHz/2Hz, and AG 4kHz/2Hz Groups. Consequently, this group (AG 1kHz/100Hz) experienced less pain and improved function.

This study had limitations. We did not assess sensory body mass index or sensory comfort during the application and did not follow up to verify the maintenance of the observed effects. In addition, although the discussion briefly mentions IFC as another modality commonly used for pain management, no direct comparisons were made between the Aussie Current and other electrical stimulation techniques. Future studies should address this limitation by evaluating the comparative effectiveness of various modalities, such as Aussie Current, TENS, and IFC, to establish their respective roles in pain control strategies. Despite these limitations, our findings suggest that the Aussie Current can be effectively applied for immediate analgesia in individuals with chronic lower back pain.

Further studies should be performed to compare additional application parameters and tests to assess functionality. In addition, we suggest that future studies include follow-ups at 24-48 h or even weeks to complement our findings and provide a more comprehensive understanding of the efficacy of the Aussie Current over different time frames.

The Aussie Current has great clinical applicability regardless of the selected parameter because it can be used for immediate analgesia before performing exercises, which favor its performance.

## CONCLUSION

The Aussie Current provides immediate analgesic effects in individuals with chronic low back pain at 1kHz frequency, but there is no consensus regarding frequency modulation.

### Implications on physiotherapy practice

The Aussie Current has great clinical applicability, regardless of the parameter selected, because it can be used for immediate analgesia before performing exercises, which favors its performance, or immediately after the session, such that any pain caused by the exercise can be alleviated.

## References

[1] Nascimento PR, Costa LO (2015). Prevalência da dor lombar no Brasil: uma revisão sistemática. Rep Public Heal.

[2] Leboeuf-Y C, Lemeunier N, Wedderkopp N, Kjaer P (2013). Evidence-based classification of low back pain in the general population: one-year data collected with SMS Track. Chiropr Man Therap.

[3] George SZ, Fritz JM, Silfies SP, Schneider MJ, Beneciuk JM, Lentz TA (2021). Interventions for the Management of Acute and Chronic Low Back Pain: Revision 2021. J Orthop Sports Phys Ther.

[4] Brech GC, Andrusaitis SF, Vitale GF, Greve JM (2012). Correlation of disability and pain with postural balance among women with chronic low back pain. Clinics (São Paulo).

[5] Khadilkar A, Odebiyi DO, Brosseau L, Wells GA (2008). Transcutaneous electrical nerve stimulation (TENS) versus placebo for chronic low-back pain. Cochrane Database Syst Rev.

[6] Resende L, Merriwether E (2018). Meta-analysis of transcutaneous electrical nerve stimulation for relief of spinal pain. Eur J Pain.

[7] Venancio RC, Pelegrini S, Gomes DQ, Nakano EY, Liebano RE (2013). Effects of carrier frequency of interferential current on pressure pain threshold and sensory comfort in humans. Arch Phys Med Rehabil.

[8] Ward AR, Oliver WG, Buccella D (2006). Wrist extensor torque production and discomfort associated with low-frequency and burst-modulated kilohertz-frequency currents. Phys Ther.

[9] Ward AR, Robertson VJ, Ioannou H (2004). The effect of duty cycle and frequency on muscle torque production using kilohertz frequency range alternating current. Med Eng Phys.

[10] Ward AR, Lucas-Toumbourou S, McCarthy B (2009). A comparison of the analgesic efficacy of medium-frequency alternating current and TENS. Physiotherapy.

[11] Ward AR, Oliver WG (2007). Comparison of the hypoalgesic efficacy of low-frequency and burst-modulated kilohertz frequency currents. Phys Ther.

[12] Imamura M, Chen J, Matsubayashi SR, Targino RA, Alfieri FM, Bueno DK (2013). Changes in pressure pain threshold in patients with chronic nonspecific low back pain. Spine.

[13] DeSantana JM, Da Silva LF, De Resende MA, Sluka KA (2009). Transcutaneous electrical nerve stimulation at both high and low frequencies activates ventrolateral periaqueductal grey to decrease mechanical hyperalgesia in arthritic rats. Neuroscience.

[14] Lisi TL, Sluka KA (2006). A new electrochemical HPLC method for analysis of enkephalins and endomorphins. J Neurosci Methods.

[15] Melzack R, Wall PD (1965). Pain mechanisms: a new theory. Science.

[16] Sluka KA, Walsh D (2003). Transcutaneous electrical nerve stimulation: basic science mechanisms and clinical effectiveness. J Pain.

[17] Silva BC, Coracini CA, Branco CL, Michelon MD, Bertolini GR (2018). Aussie current in students with chronic neck pain: a randomized controlled trial. Br J Pain.

[18] Rampazo da Silva ÉP, da Silva VR, Bernardes AS, Matuzawa FM, Liebano RE (2018). Study protocol of hypoalgesic effects of low frequency and burst-modulated alternating currents on healthy individuals. Pain Manag.

[19] Rampazo da Silva ÉP, Silva VR, Bernardes AS, Matuzawa F, Liebano RE (2019). Segmental and extrasegmental hypoalgesic effects of low-frequency pulsed current and modulated kilohertz-frequency currents in healthy subjects: randomized clinical trial. Physiother Theory Pract.

[20] Pelegrini AC, Gasoto E, Bussolaro JM, Segatti G, de Albuquerque CE, Bertolini GR (2019). The analgesic action of Aussie current in women with non-specific chronic lumbar pain. Int J Ther Rehabil.

[21] Boonstra AM, Stewart RE, Köke AJ, Oosterwijk RF, Swaan JL, Schreurs KM (2016). Cut-Off Points for Mild, Moderate, and Severe Pain on the Numeric Rating Scale for Pain in Patients with Chronic Musculoskeletal Pain: Variability and Influence of Sex and Catastrophizing. Front Psychol.

[22] Lim CY, In J (2019). Randomization in clinical studies. Korean J Anesthesiol.

[23] Almeida N, Paladini LH, Korelo RG, Liebano RE, de Macedo AC (2020). Immediate effects of the combination of interferential therapy parameters on chronic low back pain: a randomized controlled trial. Pain Pract.

[24] Jensen MP, McFarland CA (1993). Increasing the reliability and validity of pain intensity measurement in chronic pain patients. Pain.

[25] Menezes Costa LC, Maher CG, McAuley JH, Hancock MJ, de Melo Oliveira W, Azevedo DC (2011). The Brazilian-Portuguese versions of the McGill Pain Questionnaire were reproducible, valid, and responsive in patients with musculoskeletal pain. J Clin Epidemiol.

[26] Pilz B, Vasconcelos RA, Marcondes FB, Lodovichi SS, Mello W, Grossi DB (2014). A versão brasileira do STarT Back Screening Tool - tradução, adaptação cultural e confiabilidade. Braz J Phys Ther.

[27] Bohannon RW (2006). Reference values for the five-repetition sit-to-stand test: a descriptive meta-analysis of data from elders. Percept Mot Skills.

[28] Ostelo RW, Deyo RA, Stratford P, Waddell G, Croft P, Von Korff M (2008). Interpreting change scores for pain and functional status in low back pain: towards international consensus regarding minimal important change. Spine.

[29] Chou R, Huffman LH, American Pain Society; American College of Physicians (2007). Nonpharmacologic therapies for acute and chronic low back pain: a review of the evidence for an American Pain Society/American College of Physicians clinical practice guideline. Ann Intern Med.

[30] Albornoz-Cabello M, Maya-Martín J, Domínguez-Maldonado G, Espejo-Antúnez L, Heredia-Rizo AM (2017). Effect of interferential current therapy on pain perception and disability level in subjects with chronic low back pain: a randomized controlled trial. Clin Rehabil.

[31] Lara-Palomo IC, Aguilar-Ferrándiz ME, Matarán-Peñarrocha GA, Saavedra-Hernández M, Granero-Molina J, Fernández-Sola C (2013). Short-term effects of interferential current electro-massage in adults with chronic non-specific low back pain: a randomized controlled trial. Clin Rehabil.

[32] Corrêa JB, Costa LO, Oliveira NT, Lima WP, Sluka KA, Liebano RE (2016). Effects of the carrier frequency of interferential current on pain modulation and central hypersensitivity in people with chronic nonspecific low back pain: A randomized placebo-controlled trial. Eur J Pain.

[33] Almeida N, Paladini LH, Pivovarski M, Gaideski F, Korelo RI, de Macedo AC (2019). Immediate analgesic effect of 2kHz's interfering current on chronic lumbar pain: randomized clinical trial. Br J Pain.

[34] Facci LM, Nowotny JP, Tormem F, Trevisani VF (2011). Effects of transcutaneous electrical nerve stimulation (TENS) and interferential currents (IFC) in patients with nonspecific chronic low back pain: randomized clinical trial. Sao Paulo Med J.

[35] Colloca L, Tinazzi M, Recchia S, Le Pera D, Fiaschi A, Benedetti F (2008). Learning potentiates neurophysiological and behavioral placebo analgesic responses. Pain.

